# Predictors of Positive Axillary Node Clearance Following Positive Sentinel Node Biopsy: A Retrospective Analysis

**DOI:** 10.7759/cureus.104327

**Published:** 2026-02-26

**Authors:** Joseph Latham, Humayun Razzaq, Gary Osborn, Asmaa Al-Allak

**Affiliations:** 1 Urology, Royal Glamorgan Hospital, Cardiff, GBR; 2 Breast Surgery, Royal Glamorgan Hospital, Cardiff, GBR

**Keywords:** axillary node clearance, breast cancer, breast cancer metastasis, risk predictors, sentinel lymph node (sln)

## Abstract

Background

Axillary management in breast cancer is increasingly de-escalated. This study assessed whether sentinel node biopsy (SNB) histological features predict further axillary disease.

Methods

A retrospective single-centre study was performed of patients with primary breast cancer who underwent axillary node clearance (ANC) following a positive SNB. Sentinel node deposit size and extracapsular spread were analysed using t-tests and chi-square testing. Logistic regression with receiver operating characteristic analysis was used to evaluate deposit size as a predictor of positive ANC.

Results

Forty-two patients were included, of whom 23 (55%) had further nodal metastases. Larger SN deposits were associated with positive ANC (p=0.020). Logistic regression identified deposits <3 mm as predictive of no further axillary disease. Extracapsular spread was also significant (p=0.042).

Conclusions

These findings support selective axillary de-escalation, although validation in larger prospective studies is required.

## Introduction

Breast cancer is the most common cancer worldwide and is the most common cause of cancer death in women, with around 2.26 million new cases and 685,000 deaths in 2020. [1] In recent years, there has been a major shift in breast cancer surgery away from complete axillary node clearance (ANC) toward more conservative approaches. Large trials such as AMAROS, IBCSG 23-01, ACOSOG Z0011, SENOMAC, and OTOASOR increasingly support de-escalation strategies for managing the axilla [2-6].

Historically, ANC was standard for decades, causing significant morbidity, including lymphedema (up to 31%), sensory loss, and arm dysfunction in 38% of patients [7]. Sentinel node biopsy (SNB) replaced ANC as standard staging in clinically node-negative disease, reducing morbidity [8]. Now, de-escalation focuses on identifying low-risk patients who can omit or reduce axillary treatment, improving quality of life while maintaining oncologic control [9].

This study aimed to examine the histological characteristics of SNB, specifically extracapsular spread and SN deposit size, to determine whether they can predict the likelihood of further axillary node metastases and contribute to the de-escalation of axillary management.

## Materials and methods

**Figure 1:**
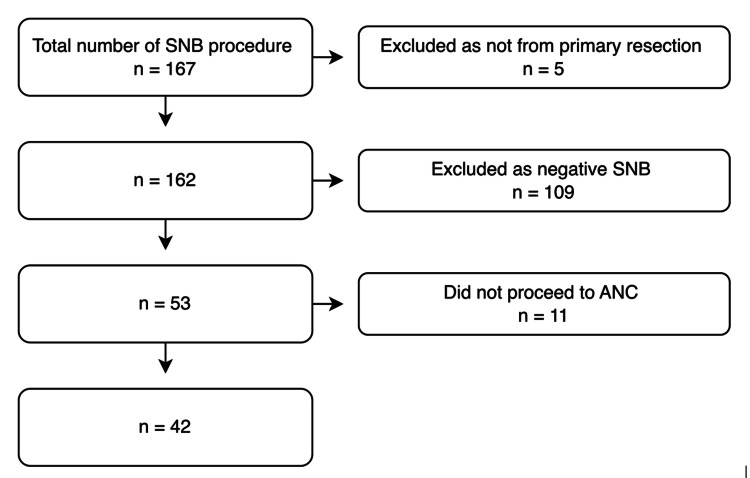
Flow diagram showing case numbers and exclusion criteria ANC: axillary node clearance, SNB: sentinel node biopsy

Retrospective data were collected from operative records and histology reports over two years. Inclusion criteria were all cases of primary breast cancer with a positive SNB that subsequently underwent ANC. Patients with recurrent disease, clinically palpable nodes, and bilateral breast cancer, those who are SN-negative, those who did not undergo subsequent ANC, or patients with incomplete records were excluded. A total of 42 cases were identified as suitable for analysis (Figure 1). SNB were conventional histology, not frozen sections.

Data collection

Data was obtained from histology slides from SNB and ANC. This provided data including demographics, presentation type (symptomatic or screening pathway), tumor type, primary tumor size, tumor grade, lymph vascular invasion, and ER/PR/HER2 status. Specific lymph node data included the number of SLN removed, the number of SLN positive, SLN extracapsular spread, SLN largest deposit size, the number of LN in ANC, the number of positive LN in ANC, AN extracapsular spread, and the largest AN deposit. The focus was on the correlation and predictive power between SNB histological characteristics and further AN involvement.

Statistical analysis

Statistical analyses were performed using GraphPad Prism software (Dotmatics, Boston, MA). We used a t-test to compare the mean sizes of the largest SN deposits between patients with positive and negative ANC. A chi-square test was used to evaluate the association between the presence of extracapsular spread and positive ANC. Statistical significance was set at p<0.05. Logistic regression was performed to identify the potential cut-off size for SN deposit size as a predictive indicator of positive ANC.

## Results

Sample characteristics

The mean age at diagnosis was 59 years (range: 40-79, median: 57). Nine of 42 cases were detected through the national screening program, with the remainder referred via the two-week-wait pathway. Histology was predominantly ductal (30/42), with 11 lobular and one micropapillary tumor. The mean tumor size was 40 mm (range: 6-180 mm, median: 28 mm). The median tumor grade was 2. ER/PR and HER2 status were incompletely recorded and therefore not analyzed.

Lymph node analysis

Of the 42 patients who underwent ANC following a positive SNB, 23 (55%) exhibited further positive nodes, defined as positive ANC, including micrometastases (<2 mm) and macrometastases (>2 mm).

Deposit site

**Figure 2:**
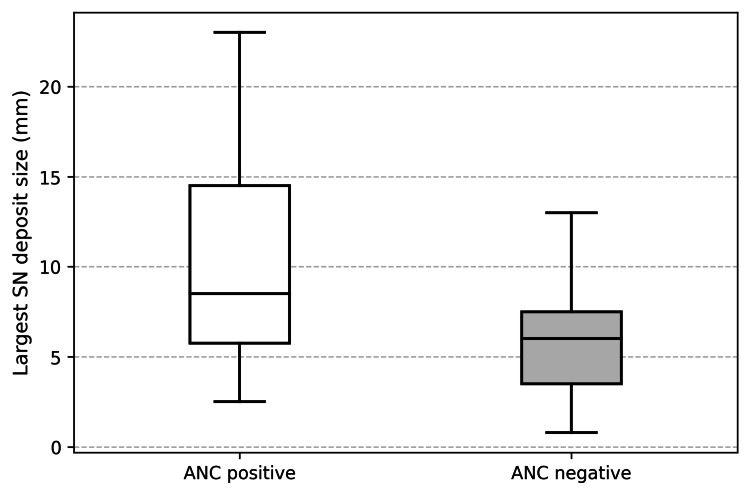
Comparing the mean largest SN deposit between ANC-positive and ANC-negative groups ANC: axillary node clearance, SN: sentinel node

The SN deposit size was found to be a significant indicator of further positive SN in ANC. The mean largest SN deposit size was significantly greater in the ANC-positive group compared with the ANC-negative group (9.75 mm versus 5.93 mm, Welch’s t-test, t=2.45, df=30.85, p=0.020) (Figure 2).

**Figure 3:**
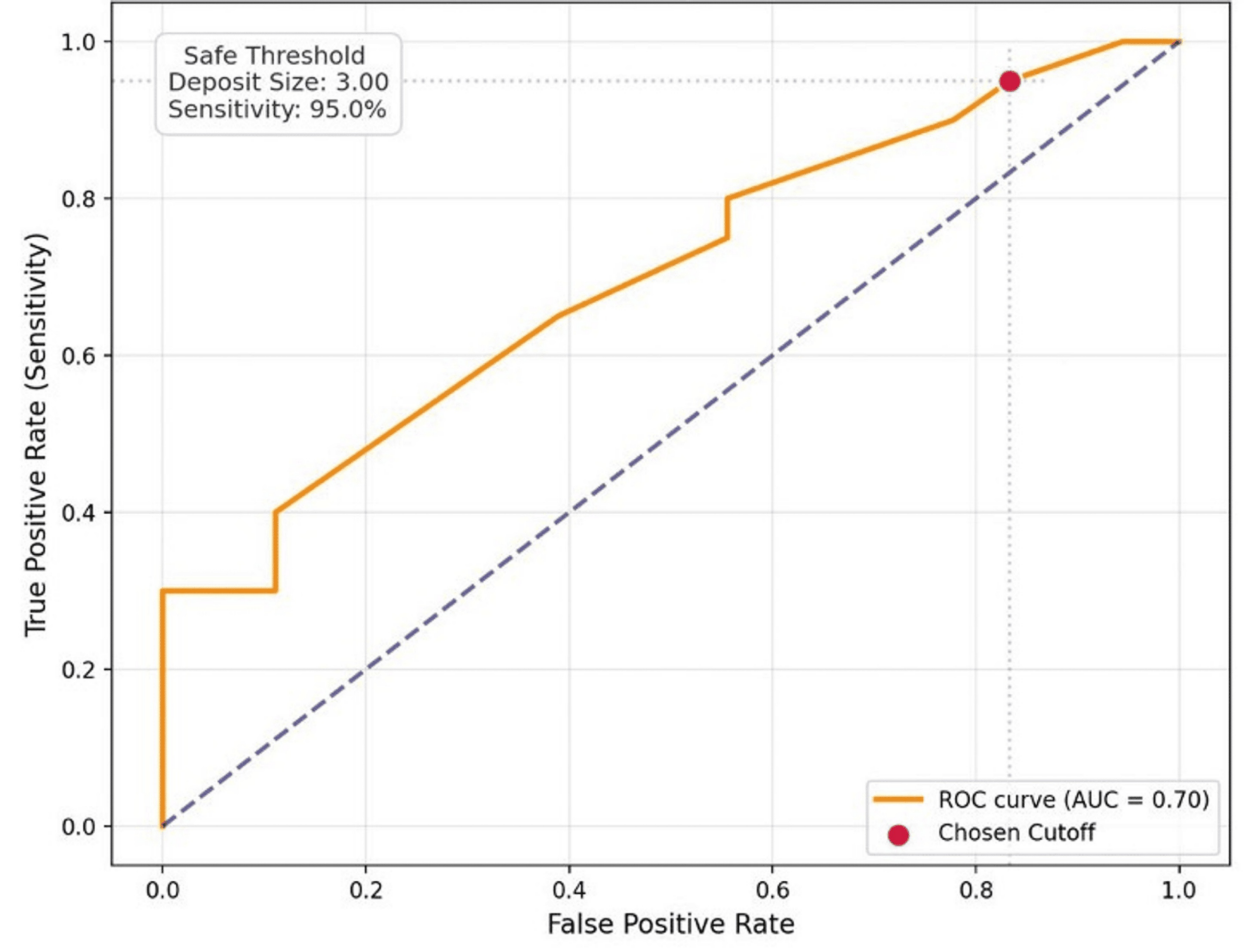
Logistic regression with ROC analysis of SN deposit size and probability of further AN disease AN: axillary node, ROC: receiver operating characteristic, SN: sentinel node

Logistic regression was performed on this data set to see if a threshold could be identified to suggest a cut-off size indicating no further axillary disease. This was found to be at <3 mm; there was a 95% probability that there would be no further AN disease with an ROC curve of 0.70 (Figure 3). However, it is associated with an 83% false positive rate (Figure 3).

Extracapsular spread

**Figure 4:**
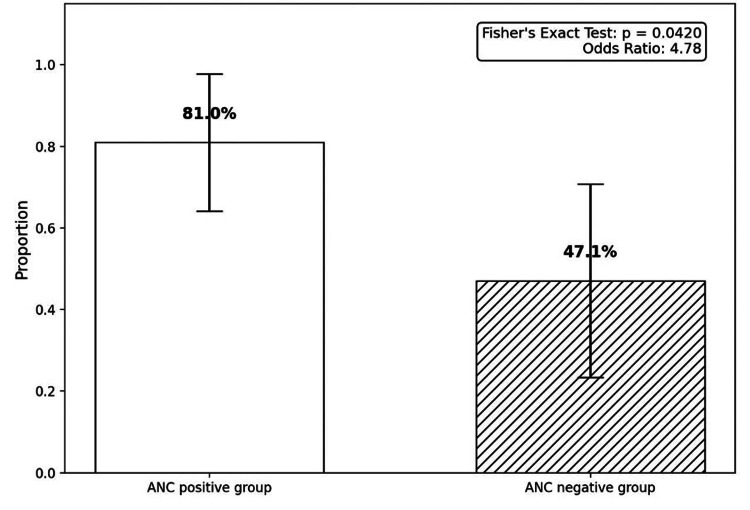
Comparing the presence of extracapsular spread between ANC-positive and ANC-negative groups (95% CI) ANC: axillary node clearance, CI: confidence interval

Extracapsular spread was also a significant predictor of positive ANC. Among patients with positive ANC, a higher proportion had extracapsular spread than those with negative ANC (χ² test, p = 0.042), with an odds ratio of 4.78.). This indicated that the extracapsular extension of tumour cells from the sentinel node predicted the likelihood of further axillary disease (Figure 4).

## Discussion

Our results

Sentinel lymph node biopsy is the standard of care for axillary node staging in patients with early breast cancer [7,8]; therefore, it is important to analyze the information it provides us to help guide clinical decisions. Our results align with current literature supporting de-escalation strategies for the axilla. We demonstrate that extracapsular spread of SN and deposit size are significant prognostic indicators for further axillary disease [7,10]. Specifically, an SN deposit of <3 mm has a 95% sensitivity that there will be no further axillary nodes. This is a potentially clinically useful histological prognostic indicator when combined with other factors.

SN-negative cancers

In SLN-negative breast cancer, studies have shown that if the SLN is uninvolved, it is highly probable that the other axillary nodes are disease-free, allowing patients to be spared from ANC [7,8]. Randomized trials such as SOUND and INSEMA have investigated the omission of axillary surgery in patients with clinically node-negative breast cancer undergoing breast-conserving surgery, finding non-inferiority compared to SNB [8,9]. Specifically, the SOUND trial found that omitting axillary surgery was non-inferior to SNB in patients with small breast cancer (<2 cm) [8].

Similarly, the INSEMA trial, with a median follow-up of six years, also concluded that the omission of surgical axillary staging was non-inferior to SNB for patients with clinically node-negative, T1 or T2 invasive breast cancer. The five-year invasive disease-free survival rate was comparable between groups, and patients in the surgery-omission group had a lower incidence of lymphedema, greater arm mobility, and less pain [9].

SN-positive cancers

In SN-positive breast cancer, the management of the axilla is variable, and ANC can be avoided only in specific circumstances. The AMAROS trial demonstrated that patients with SN micrometastases do not require ANC and can instead be treated with axillary radiotherapy alone. Five-year disease-free survival was non-inferior without ANC (87.8%) compared with ANC (84.4%), with significantly lower rates of lymphedema and neuropathy [2]. This was supported by the OTOASOR trial, which showed that axillary radiotherapy provides comparable locoregional control to ANC with significantly less morbidity [6].

Similarly, the ACOSOG Z0011 trial showed that patients with T1-T2 tumors, clinically node-negative disease, and 1-2 positive sentinel nodes receiving breast-conserving surgery and whole-breast radiation can omit ANC without compromising 10-year overall survival [4]. Likewise, the SENOMAC trial demonstrated that omitting ANC was non-inferior to completion of ANC in patients with 1-2 sentinel nodes (macrometastases) who received adjuvant systemic treatment and radiation therapy [5].

Samoilova et al. showed that patients with sentinel node deposits ≤ 5 mm (all with ≤3 positive nodes, 95% sentinel node-only, and 91% single-node involvement) have minimal risk of extensive axillary disease and may benefit from a conservative approach [7]. Additionally, the IBCSG trial shows that patients with micrometastases (≤2 mm) have even lower rates of additional nodal involvement compared to macrometastases, supporting the omission of ANC in this population [3].

Clinical implications

In the current climate of de-escalation of axillary disease, our findings provide an additional metric to support the de-escalation of treatment of the axilla. Our findings show that if the SNB demonstrated no extracapsular spread or is <3 mm, then it is highly unlikely that there will be further axillary nodal disease. This adds to the growing literature supporting the de-escalation of axillary treatment in specific groups.

Limitations

Limitations of this study include its single-center, retrospective design and small sample size. Methodologically, we were limited by the absence of detailed information on the specific staining techniques used, and a comprehensive analysis of tumor characteristics was not possible. Furthermore, given the low case numbers, our findings should be regarded as those of a pilot study, and the conclusions should therefore be interpreted with caution, considering these methodological limitations.

## Conclusions

Our study contributes to the growing literature to shift away from invasive management of the axilla. We demonstrated that an SN deposit size of <3 mm is likely to have no further AN disease on clearance. However, this should be interpreted with caution as this study is single-center and retrospective, with a small sample size, and further larger analysis is required.

## References

[1] (2026). World Cancer Research Fund: Breast cancer statistics. https://www.wcrf.org/preventing-cancer/cancer-statistics/breast-cancer-statistics/.

[2] Donker M, van Tienhoven G, Straver ME (2014). Radiotherapy or surgery of the axilla after a positive sentinel node in breast cancer (EORTC 10981-22023 AMAROS): a randomised, multicentre, open-label, phase 3 non-inferiority trial. Lancet Oncol.

[3] Galimberti V, Cole BF, Zurrida S (2013). Axillary dissection versus no axillary dissection in patients with sentinel-node micrometastases (IBCSG 23-01): a phase 3 randomised controlled trial. Lancet Oncol.

[4] Giuliano AE, Ballman KV, McCall L (2017). Effect of axillary dissection vs no axillary dissection on 10-year overall survival among women with invasive breast cancer and sentinel node metastasis: the ACOSOG Z0011 (Alliance) randomized clinical trial. JAMA.

[5] de Boniface J, Filtenborg Tvedskov T, Rydén L (2024). Omitting axillary dissection in breast cancer with sentinel-node metastases. N Engl J Med.

[6] Sávolt Á, Péley G, Polgár C (2017). Eight-year follow up result of the OTOASOR trial: The Optimal Treatment Of the Axilla - Surgery Or Radiotherapy after positive sentinel lymph node biopsy in early-stage breast cancer: a randomized, single centre, phase III, non-inferiority trial. Eur J Surg Oncol.

[7] Samoilova E, Davis JT, Hinson J (2007). Size of sentinel node tumor deposits and extent of axillary lymph node involvement: which breast cancer patients may benefit from less aggressive axillary dissections?. Ann Surg Oncol.

[8] Gentilini OD, Botteri E, Sangalli C (2023). Sentinel lymph node biopsy vs no axillary surgery in patients with small breast cancer and negative results on ultrasonography of axillary lymph nodes: the SOUND randomized clinical trial. JAMA Oncol.

[9] Reimer T, Stachs A, Veselinovic K (2025). Axillary surgery in breast cancer - primary results of the INSEMA trial. N Engl J Med.

[10] Rosen J, Manley LR, Patel A, Gandamihardja T, Rao A (2023). Prediction of negative axillary node clearance by sentinel node-positive to total node ratio: a retrospective cohort study. Ann Med Surg (Lond).

